# Quality Improvement Methodology to Optimize Safe Early Mobility in a Pediatric Intensive Care Unit

**DOI:** 10.1097/pq9.0000000000000369

**Published:** 2020-12-28

**Authors:** Neha Gupta, Amber Sones, Maegan Powell, Johanna Robbins, Stephanie Wilson, Amy Hill, Christy Thomas, Sara Ledbetter, Anne Grace Schmidtke, Chrystal Rutledge, Leslie Hayes

**Affiliations:** From the * Division of Pediatric Critical Care, University of Alabama at Birmingham, Birmingham, Ala.;; † Department of Physical Therapy, Children’s of Alabama, Birmingham, Ala.;; ‡ Department of Occupational Therapy, Children’s of Alabama, Birmingham, Ala.;; § Department of Respiratory Therapy, Children’s of Alabama, Birmingham, Ala.; and; ¶ Department of Nursing, Children’s of Alabama, Birmingham, Ala.

## Abstract

Utilization of robust quality improvement methodology in conjunction with traditional interventions to enhance an Early Mobility program (EMP) in a tertiary pediatric intensive care unit (PICU).

**Methods:**

EMP was implemented in our PICU in May 2017. The percentage of appropriate physical and occupational therapist consults were determined. We also evaluated the activity levels received by the patient and the levels for which they qualified based on their medical condition. Failure Modes and Effects Analysis (FMEA) was performed to identify potential complications related to the mobilization of critically ill children. We created 4 simulation scenarios based on FMEA prioritized results.

**Results:**

After the implementation of EMP, appropriate physical and occupational therapist consults significantly increased (*P* < 0.0001). However, most patients still failed to receive the optimal level of activity recommended by protocol. This failure was partly due to concern for safety events during mobilization. FMEA identified vital sign changes [Risk Priority Number (RPN) 97.8], staff injury (RPN 64), and pain/anxiety (RPN 60.5) as potential safety events. We performed various in-situ simulation sessions based on these potential events. In post-simulation evaluations, 100% of participants agreed that the simulation experience would improve their performance in the actual clinical setting. Feedback from simulations led to the development of an EM patient safety checklist and clinical pathway.

**Conclusions:**

We describe a novel technique of using FMEA to develop scenarios that simulate potential adverse events to optimize safe EM in PICU. An EM checklist and pathway can guide in the implementation of safe EMP.

## Abstract 2

Supplemental Digital Content is available in the text.

## INTRODUCTION

Prevention of long-term consequences of prolonged sedation and immobility has focused on providing optimal care in intensive care units (ICU). These include a decrease in critical illness-related myopathy as well as polyneuropathy and impaired pulmonary capacity. In a recent study among children, recovery to baseline function was only 28% by 3 months and 42% by 6 months following a critical illness; another study demonstrated that 45% of survivors with pediatric ICU (PICU) admission older than 28 days had unfavorable outcomes including moderate-severe disability or death.^1,2^ Early mobility (EM) has shown to improve several of these adverse effects, including muscular strength, reduced duration of delirium, hospital and ICU length of stay, and better quality of life.^3–7^

Adult ICUs have been early adopters of EM programs (EMPs). With the benefits seen with the use of such programs in adults, PICUs across the nation have started following the example set by their adult counterparts.^8^ Early mobility guidelines have been developed for critically ill children and are implemented in various institutions.^8–12^ Wieczorek et al. developed similar guidelines and recommended different activity levels based on their patients’ clinical status in the PICU.^8^ Cuello-Garcia et al. described various barriers to EM, including limited resources, patient factors (for example, level of sedation and ability to cooperate), and staff and caregiver anxiety.^10^ Most studies have shown that safe implementation of EM in the PICU is feasible, with a systematic review reporting no adverse events after the implementation of active and passive mobilization.^9,10^ Given the safety data of these protocols, the implementation of EM in PICUs continues to rise.^11^ Still, the efficacy of rehabilitation in the pediatric population remains undetermined at this time.^10^

We describe our experience using robust quality improvement and patient safety methodology to identify challenges and barriers with the implementation of an EMP in a tertiary care PICU. This project’s goal was to utilize robust quality improvement (QI) methodology in conjunction with traditional interventions to enhance our EMP.

## METHODS

This report is a single-center quality improvement project conducted at a large tertiary care pediatric hospital from May 2017 to July 2019. Our PICU is a high-acuity, 24-bed unit in an institution with a separate intermediate care unit. This project’s aims included developing an EMP, developing and implementing the EM guidelines, assessing the barriers to EM using Failure Modes and Effects Analysis (FMEA), and using simulation to overcome those barriers.

### Development of an EMP

We defined EM as “implementation of therapeutic interventions aimed at mobilization within the first 72 hours of PICU admission.”^13^ To meet these criteria, an activity level had to be assigned, physical therapy (PT)/occupational therapy (OT) consult had to be placed, and an intervention by the physical or occupational therapist must be performed within 72 hours of PICU admission. The PICU EM team consisted of critical care physicians, physical therapists, occupational therapists, respiratory therapists (RTs), a child life specialist, nurses, and our Virtual Pediatric Systems, LLC database team. All the team members attended evidence-based health care continuing education seminars focusing on EM in critically ill children.

### Implementation of EM Guidelines

Our group adapted EM Guidelines from Wieczorek et al.^8^ and implemented these in our PICU in May 2017. Our guidelines consist of criteria for mobilization of critically ill children based on their severity of illness with specific therapeutic interventions for each level (Table 1). All patients admitted to the PICU requiring mechanical ventilation received PT/OT consults on PICU day 3 if they remained on the ventilator. The EM team chose day 3 for 2 reasons: 1. this would eliminate consults on very short-stay patients, and 2. would allow time for clinical stabilization. Providers could consult PT/OT before day 3 if needed.

**Table 1.** Early Mobility Guidelines—Patient Activity levels Based on the Severity of Illness

Levels	Level 0	Level 1	Level 2	Level 3
Criteria for levels	Not stable for ROM or stimulation (hemodynamically unstable patients requiring active resuscitation)	Intubated, FiO_2_ ≥ 60%	Intubated, FiO_2_ < 60%	External ventricular drain cleared by neurosurgery
	*PT/OT consulted in anticipation of future therapy needs	Intubated PEEP ≥ 8	Intubated PEEP < 8	Baseline pulmonary support
		Oscillator	Renal replacement therapy if not femoral access	Noninvasive respiratory support with FiO_2_ < 60%
		Extracorporeal membrane oxygenator	Arterial line (any location)	SBS −1 to +3
		Critical airway	Chest tube	
		Vasoactive medications other than milrinone	New tracheostomy after ties changed if not critical airway	
		Femoral access	O_2_ saturation > 92%	
		Acute spinal cord injury or severe traumatic brain injury (<7 days)	SBS −1 to +3	
		Sedated and SBS −3 to −2		
Therapeutic interventions	PT/OT	PT/OT	PT/OT	PT/OT
	Issue appropriate splints PRN	ROM, splinting	Level 1 activities plus	Level 1 and 2 activities plus
	Daily check-ins with team	In-bed strengthening	Bed in chair position	Out-of-bed to chair
		Recommendations for positioning	Consider edge of bed sitting	Out-of-bed strengthening
		Positive touch for infants, toddlers	Consider out-of-bed transfer	Ambulation
			Consider ambulation unless arterial line in place	
		Nurses	Nurses	Nurses
		Skin risk assessment	Skin risk assessment	Activities of daily living
				Bedside commode
		SLP	SLP	SLP
		Assess for communication difficulties	Assess for communication difficulties	Assess swallowing

PEEP indicates positive end-expiratory pressure; PRN, Pro Re Nata; ROM, range of motion; SBS, State Behavioral Score (sedation assessment tool used for pediatric patients to monitor sedation level in pediatric critical care unit)^21^; SLP, Speech-Language Pathologist.

The percentage of appropriate daily PT/OT consults (defined as ordered by the third day of mechanical ventilation) were determined before and after the initial implementation of our EMP. We compared the percentages 4 months pre-implementation (January-April 2017) with 4 months post-implementation (January-April 2018) using Shewhart control charts. Data were collected during similar months to prevent bias from seasonal variation in diagnoses. We also evaluated the activity levels for which patients qualified based on their medical condition and the activity levels received by the patient, using the criteria in Table 1. Minitab 18™ (Minitab, LLC. State College, PA) was used for statistical analysis.

### Challenges During the Implementation of EM Guidelines

After the implementation of EM in our PICU, perceived barriers and safety concerns existed among providers and caregivers, including the risk of unplanned extubation, device dislodgement, and significant vital sign changes. Even though the literature indicates a low rate of these adverse events, staff must be well prepared for them.^12^ Additionally, there was variation in daily emphasis on EM among the PICU providers, especially for optimizing activity levels. We identified these root causes for less than optimal activity levels as the most critical gaps in performance. As a result, we employed quality improvement and patient safety tools to address these leverage points for improvement.

### Failure Modes and Effects Analysis

We performed an FMEA among our interprofessional unit-based QI committee to address the safety concerns about EM. This committee consists of the EM team members, additional critical care attendings and fellows, a pediatric psychiatrist, a pediatric rehabilitation medicine attending, other nurses and RTs including nursing and RT leaders. FMEA is a proactive risk assessment tool used to evaluate the severity, occurrence, and detection of risks and prioritize them based on urgency.^14^ Our FMEA began with a review of the detailed process of mobilizing critically ill children across different activity levels. We performed a hazard analysis by recording potential EM-related adverse events and their effects, assigning severity, occurrence, and detectability scores (each ranging from 1 to 10) for each adverse event and identifying actions required. The team performed the FMEA to avoid any adverse events in our unit. Each member of the committee assigned severity, occurrence, and detectability scores, and those scores were averaged for each failure mode. We modified the FMEA by not specifically outlining effects for each step since the effect of an adverse event in EM is patient deterioration. Risk Priority Numbers (RPNs) were calculated for each risk or event by:


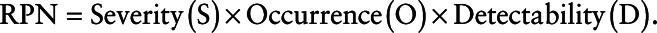


RPNs were then rank-ordered to determine priority. Typically, a higher RPN for an event potentially indicates a higher priority to create a new process to prevent it. We used FMEA to identify the potential severity, frequency, and detectability of possible complications related to the mobilization of critically ill children.

### Challenges of Using FMEA

Performing an FMEA involved various challenges, including difficulties in defining the failure modes and causes and developing the rating scales for severity, occurrence, and detectability. These ratings varied with the profession of individual team members. Therefore, we used the average of the individual ratings.

### EM Simulations

The team created 4 simulation scenarios based on the FMEA results and literature review. Scenarios included:

1. Vital sign changes resulting in a patient fall (scenario 1);
2. Unplanned extubation/dislodged tracheostomy tube (scenario 2);
3. Staff injury due to patient anxiety/delirium (scenario 3); and
4. Cardiorespiratory arrest (scenario 4).

In situ simulations were conducted in our PICU. Each simulation included 4 nurses, 2 RTs, and a PT, OT, nurse practitioner, pediatric resident, and critical care fellow. The cases began with only a nurse, RT, PT, and OT mobilizing the patient, to simulate a real-life scenario. Depending on the activity level prescribed and adverse event that occurred, these 4 participants could call other participants for help. We used either high fidelity mannequins or trained actors as appropriate for each scenario. For example, standardized patients were used for scenarios 1 and 3, while a high-fidelity mannequin with a tracheostomy was used for scenario 2. For scenario 4, we began with a standardized patient; once the patient collapses, the actor was replaced with a mannequin to continue with the management of cardiorespiratory arrest. The activity levels were modified in these 4 scenarios to provide variation in the next set of simulations. For instance, for scenario 4, we conducted the simulation with the actor walking in the unit and experiencing cardiac arrest at the far end of the unit. In the next set of simulations, we changed the activity level, and the code event occurred while moving the patient out of bed to the chair. This way, we could provide variability in repeat cases and training to recognize and manage these events in different situations. Simulations occurred every 4–6 months based on the availability of participants, simulation staff and equipment, and empty patient rooms in the PICU. These simulations focused on mobilizing patients with complex conditions in a safe manner and quickly recognizing potential adverse events by the therapists, nurses, and RTs. We created standard training for bedside providers to call for additional assistance as events occurred and to manage events efficiently. Debriefing focused on medical management and process improvement opportunities. The team performed post-simulation evaluations of the knowledge, skills, and attitudes of these scenarios. These stimulations are still ongoing, with the goal of each participant completing all 4 scenarios.

### Ethics

We used guidelines for reporting quality improvement initiatives published by the SQUIRE Development Group for this manuscript.^15^ This QI project was exempt from our Institutional Review Board.

## RESULTS

**Fig. 1.**
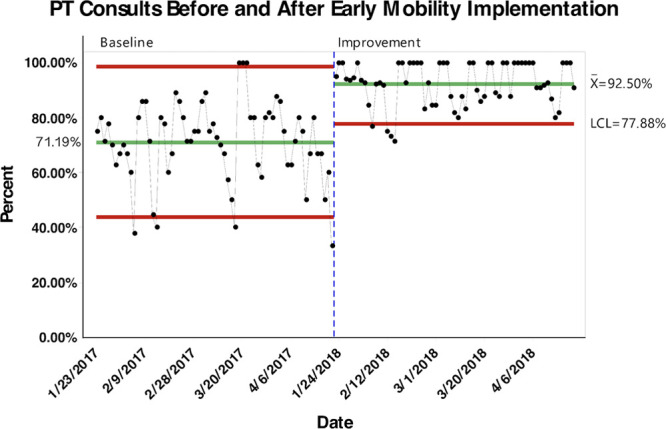
Control chart with percent of PICU patients receiving appropriate PT consults before and after implementation of early mobility guidelines. LCL indicates lower control limit.

**Fig. 2.**
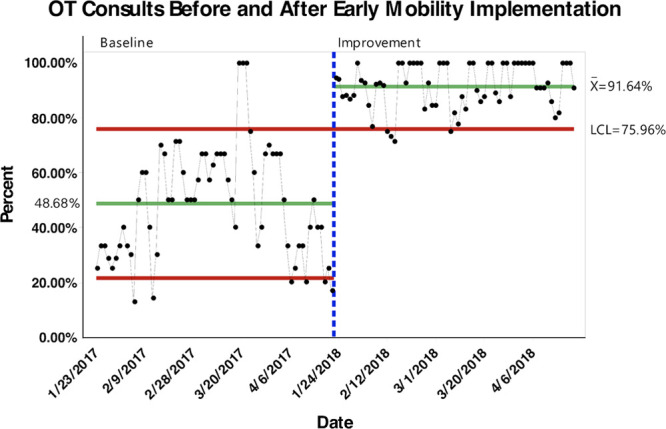
Control Chart with percent of PICU patients receiving appropriate OT consults before and after implementation of early mobility guidelines. LCL indicates lower control limit.

With initial implementation of our EMP, the percent of appropriate PT consults increased significantly from 71.2% pre-implementation to 92.5% post-implementation (*P* < 0.0001; 95% CI, −0.254 to −0.172) (Fig. 1). An even more significant increase was seen in percent of OT consults from 48.7% pre-implementation to 91.6% post-implementation (*P* < 0.0001; 95% CI, −0.485 to −0.374) (Fig. 2).

**Fig. 3.**
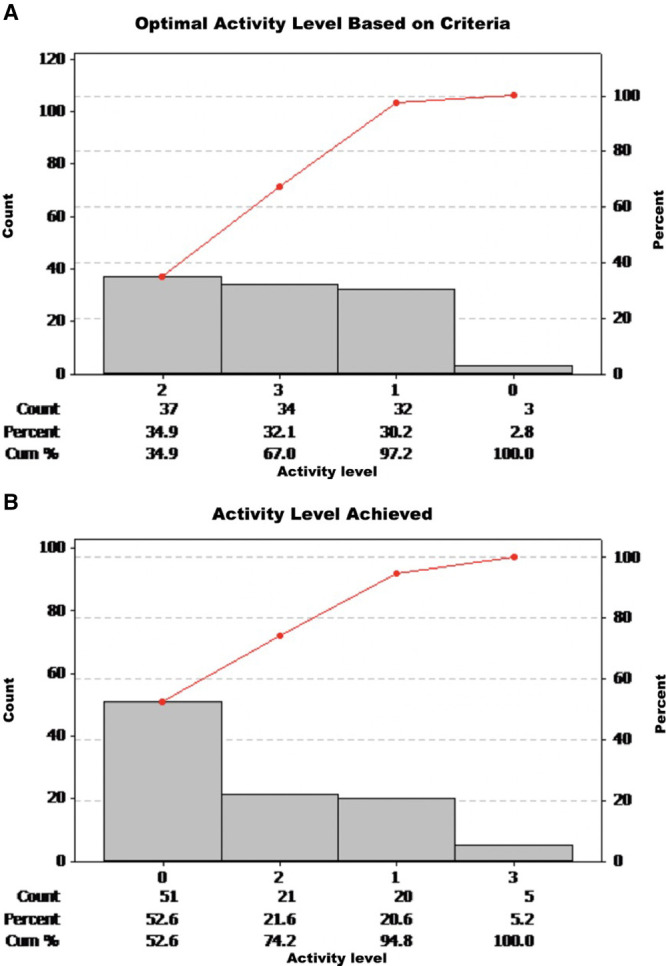
Pareto charts demonstrating. A, The frequency of activity levels PICU patients could have received based on our criteria. B, Activity levels PICU patients received.

Although a significant improvement in the percent of patients receiving PT/OT consults was seen, we identified a gap in performance between patients’ level of activity based on their medical condition and the level of activity patients received daily. The Pareto chart depicted in Figure 3A and B demonstrate the distribution of optimal activity levels that patients qualified for based on the criteria outlined above and the actual activity level achieved by them, respectively, during February and March 2019. As noted, instead of 67% of patients (n = 98), only 26.8% of patients received daily activity levels of 2 and 3. Also, while only 2.8% of patients should have received the lowest level of intervention (level 0), data reveals that 52.6% received this lowest level of intervention.

The FMEA identified potential patient and employee safety events during EM. **Appendix 1 (Supplemental Digital Content 1**, http://links.lww.com/PQ9/A221) shows the results of the FMEA. Various possible adverse events and their causes were identified based on the level of activity. Many of the failure modes overlapped for each activity level. We combined these failure modes, and Table 2 shows the simplified summary of these results. The events with the highest RPNs included vital sign changes (RPN 97.8), followed by staff injury (RPN 64) and pain/fatigue/anxiety/distress (RPN 60.5). Based on the ranking of RPNs, cardiorespiratory arrest ranked lowest on the scale (RPN 11.9). This result is an expected finding since it is considered a rare and readily detectable event. The EM team used this information to develop in situ simulation scenarios that address the identified high-risk, adverse events. Despite having the lowest RPN, it was essential to include cardiorespiratory arrest in the simulation scenarios as it was most concerning to the staff.

**Table 2.** Summary of FMEA for Potential Adverse Events During Early Mobility

Failure Modes	Severity (S)	Occurrence (O)	Detectability (D)	RPN
Vital sign changes (hypotension/desaturation)	3.8	6.6	3.9	97.8
Staff injury	8	1	8	64
Pain/fatigue/anxiety/distress	5.6	4.7	2.3	60.5
Fall	7.8	3	1.5	35.1
Equipment failure	8	3	1.3	31.2
Dislodged endotracheal/tracheostomy tube	8.7	2.1	1.3	23.8
Dislodged devices/lines	5	2.7	1.6	21.6
Staff unavailability	4.8	2.5	1.3	15.6
Pressure injury if left in chair for long time	5	3	1	15
Cardiorespiratory arrest	9.9	1.2	1	11.9

Various members of PICU staff participated in the in- situ simulations. Post-simulation evaluations showed that 100% of participants agreed that the simulation experience will improve their performance in the actual clinical setting and that the sessions were a valuable learning experience. Common themes that emerged from participant evaluations included:

1. Preparation—having all the necessary equipment in place before starting rehabilitation therapy is key to streamlining a safe process;
2. Teamwork—timely notification and presence of essential staff are important before mobilizing high-risk patients;
3. Role clarity—having clear roles and responsibilities during mobility as well as in the case of associated adverse events; and
4. Standardization of the process—an EM checklist would be helpful to review before patient participation in mobilization.

This analysis led to the development of an EM patient safety checklist and an EM clinical pathway for “out of bed” mobility. During subsequent EM simulation scenarios, we piloted the use of these tools. We modified the checklist and pathway based on the evaluations and debriefing from the subsequent set of simulations. **Appendix 2 (Supplemental Digital Content 2**, http://links.lww.com/PQ9/A222) and **Appendix 3 (Supplemental Digital Content 3**, http://links.lww.com/PQ9/A223) show the final version of the EM checklist and EM clinical pathway. We also created an EM cart with all the supplies required during EM in addition to our airway supply cart. The list of items in this cart is given in **Appendix 4 (Supplemental Digital Content 4**, http://links.lww.com/PQ9/A224).

## DISCUSSION

The results from our study show that the application of EMP in PICU is feasible. Our data show that after the initial implementation of the EM protocol in our PICU, there was a substantial increase in the percentage of appropriate PT and OT consults (92.5% and 91.6%, respectively). Most PICU providers believe that EM can decrease the length of ICU stay and mechanical ventilation and reduce the incidence of delirium.^16^ Al-qaqaa et al. reported a reduced hospital length of stay with early mobilization in the PICU^17^. In addition to critical illness myopathy and polyneuropathy, immobility can also increase the incidence of decubitus ulcers.^18^ Therefore, it is essential to mitigate the barriers and challenges to achieve optimal mobility and rehabilitation for critically ill children to improve functional outcomes.

Even though a significant increase in the number of PT/OT consults was seen, we did not optimize daily therapy activities in our patients due to staff apprehension of EM. We describe a novel technique using FMEA, a quality improvement, and patient safety tool, to identify potential adverse events during EM and develop interprofessional simulation scenarios informed by the FMEA RPNs from our staff in the PICU. These simulations helped staff become more comfortable with mobilizing critically ill patients, including those with an endotracheal or tracheostomy tube, and helped us identify the need for a patient safety checklist and a mobile EM supply cart to optimize safe mobilization. We improved and refined our EMP for patients undergoing “out of bed” mobility to include a patient safety checklist and mobilization pathway (see **Appendix 2, Supplemental Digital Content 2**, http://links.lww.com/PQ9/A222 and **Appendix 3, Supplemental Digital Content 3**, http://links.lww.com/PQ9/A223) to optimize safe mobilization and standardize care delivery during EM.

Even though literature reports a low rate of adverse events during the mobilization of children, safety concerns still exist. Joyce et al. described various perceived barriers among the PICU staff related to EM with the risk of endotracheal tube dislodgement, loss of indwelling catheters, and increased workload among the most serious concerns.^16^ Since EM in older children is perceived to be safer, they are more likely to receive rehabilitation in the PICU.^19,20^ We also recognized the potential for various adverse events, including vital sign changes, staff injury, device dislodgement, and equipment failure. It is human nature to focus on rare serious events like cardiorespiratory arrest, which are typically easily detectable. However, it is essential to prioritize events that occur more commonly with subtle presentations, like vital sign changes, which can lead to more severe complications. FMEA can not only help us identify these events, but also helps us prioritize them according to their seriousness and urgency in addressing them.

Determining safe EM interventions while considering the patient’s medical condition is imperative to a safe and feasible EMP. Establishing standard, predictable integration of activity levels based on daily medical conditions with the initial implementation of our EMP proved difficult because of provider variation in adoption. This barrier limited the level of therapeutic intervention provided to appropriate EM candidates. Our FMEA-based simulation scenarios identified inadequate staffing and readily available equipment to effectively and safely implement more aggressive EM interventions for PICU patients. Our bedside staff identified this obstacle as one of several barriers to optimal EM. Similarly, literature reports other barriers to early mobilization in the PICU, including a lack of guidelines and the inability to place physician orders in time for PT to start rehabilitation.^19^ A lack of standardized protocol or guidelines can lead to suboptimal patient mobilization.

Despite the improved comfort level of our staff with EM, we continue to face sustained obstacles in terms of limited PT/OT staffing and lack of equipment required for mobilization of patients. Identification of these barriers has also led us to prioritize our next steps. Our EM team is currently partnering with hospital and unit leadership to acquire assistive and adaptive equipment that will significantly reduce the number of staff required to mobilize a critically ill child while reducing the risk of employee injury. Acquisition of this equipment will also increase our ability to perform therapeutic interventions outside of the scheduled PT/OT sessions, including weekends, when we currently do not have access to PT/OT services. We are working towards full-time PT/OT services for our PICU patients, including weekend services and therapists’ continuity, even after patient transfer to another inpatient unit. To ensure a sustainable program with improved outcomes for our patients, we continue to evaluate additional ways to improve EM workflow within our PICU.

## LIMITATIONS

Our report is a single-center study, and our experience with the implementation of an EMP and the barriers identified may not be generalizable to every PICU. The FMEA results could have been influenced by the perceived or actual availability or lack of resources and provider beliefs at our center, which might differ at other institutions. However, proactive use of quality improvement tools like FMEA to guide and hone an individualized EM protocol can be helpful, making it a generalizable approach for all institutions.

## CONCLUSIONS

As a part of EMP implementation at our institution, we utilized robust quality improvement and patient safety methodology to create improved standard work and develop a proactive approach to avoiding potential adverse events. An EM checklist and pathway helped guide the implementation of effective and safe strategies to prevent complications during the mobilization of critically ill children. Further research is needed to evaluate the generalization of these findings to other institutions.

## IMPLICATIONS

The partnership of quality improvement, patient safety, and healthcare simulation results in a robust approach to safe care delivery in the PICU’s complex environment. An EM checklist and pathway can guide us in implementing effective and safe strategies to avoid complications during the mobilization of critically ill children.

## DISCLOSURE

The authors have no financial interest to declare in relation to the content of this article.

## ACKNOWLEDGMENTS

We thank all the Early Mobility team members for their participation in failure mode and effect analysis. We also thank all the pediatric intensive care unit staff at our institution for their assistance in simulations and providing feedback.

## Supplementary Material


