# Supplementary material for: Quality Improvement Methodology to Optimize Safe Early Mobility in a Pediatric Intensive Care Unit
Source: Pediatr Qual Saf. 2020 Dec 28;6(1):e369. doi: 10.1097/pq9.0000000000000369 (PMC7774997; doi:10.1097/pq9.0000000000000369)
Supplement: Supplementary file 1 [file pqs-6-e369-s001.pdf]

Steps in process	Failure modes	Causes	Severity (S) (1 not bad, 10 the worst)	Occurrence (O) (1 almost never, 10 all the time)	Detectability (D) (1 easy to know, 10 hard to know)	Risk Priority Number (RPN= SxOxD)	Actions
**Patient more mobile in bed**	Unplanned extubation	Tube not secured before/during EM	8.7	2.3	1.3	26	Secure tube before EM and have respiratory therapist (RT) in room during mobilization
	Fall	Lack of preventive strategies like bed rails, orthostatic hypotension	8	2	1.5	24	Take fall precautions, monitor vital signs
	Respiratory distress	Patient not stable for mobility, tube/equipment failure	4	6	1	24	Evaluate for stability and check equipment before mobilization
	Dislodged devices/lines	Devices/lines not secured before/ during EM	5.8	1.8	2.1	21.9	Secure lines/devices before mobilization and having extension tubing for lines. Stop infusions through lines if possible.
	Vital sign changes	Tachycardia, hypertension, hypotension and desaturation-patient unstable for EM, devices/lines not secured, equipment failure, pain	3	6	1	18	Evaluate for stability and pain, and check equipment and secure devices/lines before mobilization
	Cardiorespiratory arrest	Patient not stable for EM	10	1	1	10	Evaluate for stability before mobilization
	Dialysis line malfunction	Line not secured or kinking of tubing	3	3	1	9	Secure lines before mobilization and check tubing during mobilization. Briefly stop dialysis and remove tubing if possible.
**Patient sits on edge of bed**	Orthostatic hypotension	Patient suddenly sat up in bed	5	7.5	3	112.5	Stepwise slowly mobilizing to side of bed and monitor vitals
	Staff injury	Staff not careful, Patient agitated/delirious	10	1	8	80	Evaluate for delirium and ability to follow commands, staff to take precautions
	Fall	Lack of preventive strategies, orthostatic hypotension	8.3	6.7	1.2	66.7	Take fall precautions, monitor vital signs
	Dislodged devices/lines	Devices/lines not secured before/ during EM, line extension not present	6	2.7	2.5	40.5	Secure lines/devices before mobilization and having extension tubing for lines. Stop infusions through lines if possible.
	Pain	Pain medication not administered before EM	4	5	2	40	Give pain medication before mobilization

	Unplanned extubation	Tube not secured before/during EM	8.7	2.5	1	21.8	Secure tube before EM and have RT in room during mobilization
	Cardiorespiratory arrest	Patient not stable for EM	10	1	1	10	Evaluate for stability before mobilization
**Patient transfers to chair**	Orthostatic hypotension	Patient suddenly stood up	4	7	5	140	Stepwise mobilization and monitor vital signs
	Oxygen desaturation	Patient not stable for mobility, tube/equipment failure	4	4	4	64	Evaluate for stability and check equipment before mobilization
	Pain	Pain medication not administered before EM	4	7	2	56	Give pain medication before mobilization
	Fatigue	Lack of strength, altered circadian rhythm, other events during the day	2	9	3	54	Schedule EM earlier in the day, evaluate sleep pattern
	Patient left in chair too long leading to pressure injury	Nurses/therapists busy with other patients	6	5	1	30	Monitor patients while in chair, specify time for being in chair, evaluate for pressure injury
	Fall	Lack of preventive strategies, orthostatic hypotension	7.7	2.1	1.3	21	Take fall precautions, monitor vital signs
	Unplanned extubation	Tube not secured before/during EM	8.4	2.5	1	21	Secure tube before EM and have RT in room during mobilization
	Dislodged devices/lines	Devices/lines not secured before/ during EM, line extension not present	5.3	3.3	1	17.5	Secure lines/devices before mobilization and having extension tubing for lines. Stop infusions through lines if possible.
	Cardiorespiratory arrest	Patient not stable for EM	10	1	1	10	Evaluate for stability before mobilization
**Patient ambulating inside room**	Orthostatic hypotension	Patient suddenly stood up	4	7	5	140	Stepwise mobilization and monitor vital signs
	Respiratory distress	Patient not stable for mobility, tube/equipment failure	8	3	3	72	Evaluate for stability and check equipment before mobilization
	Dislodged devices/lines	Devices/lines not secured before/ during EM, line extension not present	5.7	4.5	2.3	59	Secure lines/devices before mobilization and having extension tubing for lines. Stop infusions through lines if possible.
	Unplanned extubation	Tube not secured before/during EM	6.3	3.5	2.3	50.7	Secure tube before EM and have RT in room during mobilization

	Staff injury	Staff not careful, Patient agitated/delirious	6	1	8	48	Evaluate for delirium and ability to follow commands, staff to take precautions
	Fall	Lack of preventive strategies, orthostatic hypotension	7	2.5	2	35	Take fall precautions, monitor vital signs
	Cardiorespiratory arrest	Patient not stable for EM	10	1	1	10	Evaluate for stability before mobilization
	Equipment failure	Leads came off the patient, extension cord not working, transport monitor not working, ventilator failure	7	1	1	7	Check equipment before mobility and have extension cords ready before mobility
**Patient in wheelchair outside of room**	Oxygen desaturation	Patient not stable for mobility, tube/equipment failure	2	8	7	112	Evaluate for stability and check equipment before mobilization
	Equipment failure	Leads came off the patient, extension cord not working, transport monitor not working, ventilator failure	7	7	2	98	Check equipment before mobility and have extension cords ready before mobility
	Pain/delirium/anxiety	Pain medication not administered before EM, presence of delirium/anxiety	4.5	5	2	45	Give pain medication before mobilization, evaluate and treat anxiety and delirium
	Staff availability	Staff busy with other patients, staff shortage	5.5	4	1.5	33	Schedule mobility when nurses, RT and PT/OT available
	Respiratory distress	Patient not stable for mobility, tube/equipment failure	7.5	2.2	1.2	32	Evaluate for stability and check equipment before mobilization
	Fall	Lack of preventive strategies, orthostatic hypotension	7.5	2.2	1.2	19.8	Take fall precautions, monitor vital signs
	Cardiorespiratory arrest	Patient not stable for EM	8	2	2	14.7	Evaluate for stability before mobilization
	Unplanned extubation	Tube not secured before/during EM	10	1	1	10	Secure tube before EM and have RT in room during mobilization
	Dislodged devices/lines	Devices/lines not secured before/ during EM, line extension not present	4.5	1	1	4.5	Secure lines/devices before mobilization and having extension tubing for lines. Stop infusions through lines if possible.

	Patient left in chair too long leading to pressure injury	Nurses/therapists got busy with other patients	4	1	1	4	Monitor patients while in chair, specify time for being in chair, evaluate for pressure injury
**Patient ambulating outside of room**	Vital sign changes	Tachycardia, hypertension, hypotension and desaturation- patient unstable for EM, devices/lines not secured, equipment failure, pain	4	7	5	140	Evaluate for stability and pain, and check equipment and secure devices/lines before mobilization
	Staff injury	Staff not careful, Patient agitated/delirious	8	1	8	64	Evaluate for delirium and ability to follow commands, staff to take precautions
	Respiratory distress	Patient not stable for mobility, tube/equipment failure	8	3	2	48	Evaluate for stability and check equipment before mobilization
	Fall	Lack of preventive strategies, orthostatic hypotension	8.4	2.7	1.7	38.6	Take fall precautions, monitor vital signs
	Cardiorespiratory arrest	Patient not stable for EM	9.8	1.5	1	14.7	Evaluate for stability before mobilization
	Dislodged devices/lines	Devices/lines not secured before/ during EM, line extension not present	4.7	3	1	14.1	Secure lines/devices before mobilization and having extension tubing for lines. Stop infusions through lines if possible.
	Unplanned extubation	Tube not secured before/during EM	10	1	1	10	Secure tube before EM and have RT in room during mobilization
	Equipment failure	Leads came off the patient, extension cord not working, transport monitor not working, ventilator failure	10	1	1	10	Check equipment before mobility and have extension cords ready before mobility
	Staff availability	Staff busy with other patients, staff shortage	4	1	1	4	Schedule mobility when nurses, RT and PT/OT available
