# Supplementary material for: Quality Improvement Methodology to Optimize Safe Early Mobility in a Pediatric Intensive Care Unit
Source: Pediatr Qual Saf. 2020 Dec 28;6(1):e369. doi: 10.1097/pq9.0000000000000369 (PMC7774997; doi:10.1097/pq9.0000000000000369)
Supplement: Supplementary file 2 [file pqs-6-e369-s002.pdf]

Early Mobility Patient Safety Checklist

[To be conducted by PICU attending or fellow (MD)/nurse practitioner (NP), bedside nurse, respiratory therapist, physical and occupational therapist]

Please mark √ for Yes and X if not applicable. If answer to any of the following questions is NO at any time, do not proceed with the procedure or stop the procedure.

- ☐ Patient is stable for early mobility (stable vital signs)
- ☐ Patient agrees that he/she is ready for mobility
- ☐ All staff members assisting are comfortable with mobility

PICU MD/NP

- ☐ PICU MD/NP is aware of the procedure
- ☐ Early Mobility activity level specified by MD/NP ______

Bedside nurse

- ☐ All medical lines (central line, arterial line, vascular catheter) are secured and extension tubing is available if needed
- ☐ Portable monitor with pulse oximetry is present if patient is getting out of bed
- ☐ Feeds, fluids and medications are paused and disconnected after talking with MD/NP
- ☐ Extension tubing is present if getting out of bed and there is need to continue medications/fluids

Respiratory therapist

Check all necessary equipment that apply:

In room mobility:

- ☐ Suction equipment including suction catheter kits
- ☐ Suction extension tubing
- ☐ Ambubag and mask
- ☐ Oxygen extension tubing if on oxygen
- ☐ Stocked portable 'Go Bag' for patients with tracheostomy
- ☐ Stocked Early Mobility cart outside the room
- ☐ Stethoscope

Out of room mobility:

- ☐ Portable suction
- ☐ Portable oxygen tanks appropriate for the duration of mobility
- ☐ Stocked portable 'Go Bag' for patients with tracheostomy
- ☐ Ambubag and mask
- ☐ Stethoscope

Physical/Occupational therapist

Check all necessary equipment that apply:

In room mobility:

- ☐ Draw sheet
- ☐ Wheelchair/Specialty chair
- ☐ Moto Med: In-Bed Arm/Leg Stationary Bicycle
- ☐ Tilt Table
- ☐ Moveo Squat Table
- ☐ TruRize Specialty Chair
- ☐ Cycle

Out of room mobility:

- ☐ Draw sheet/Backboard
- ☐ Wheelchair/Specialty chair
- ☐ Standard rolling walker or Eva walker
- ☐ Gait belt (if tolerated with placement of lines/tubes)
- ☐ Standard rolling walker or Eva walker
