# Supplementary figures and images for: Quality Improvement Methodology to Optimize Safe Early Mobility in a Pediatric Intensive Care Unit
Source: Pediatr Qual Saf. 2020 Dec 28;6(1):e369. doi: 10.1097/pq9.0000000000000369 (PMC7774997; doi:10.1097/pq9.0000000000000369)

## Out of Bed Mobilization of patients in PICU

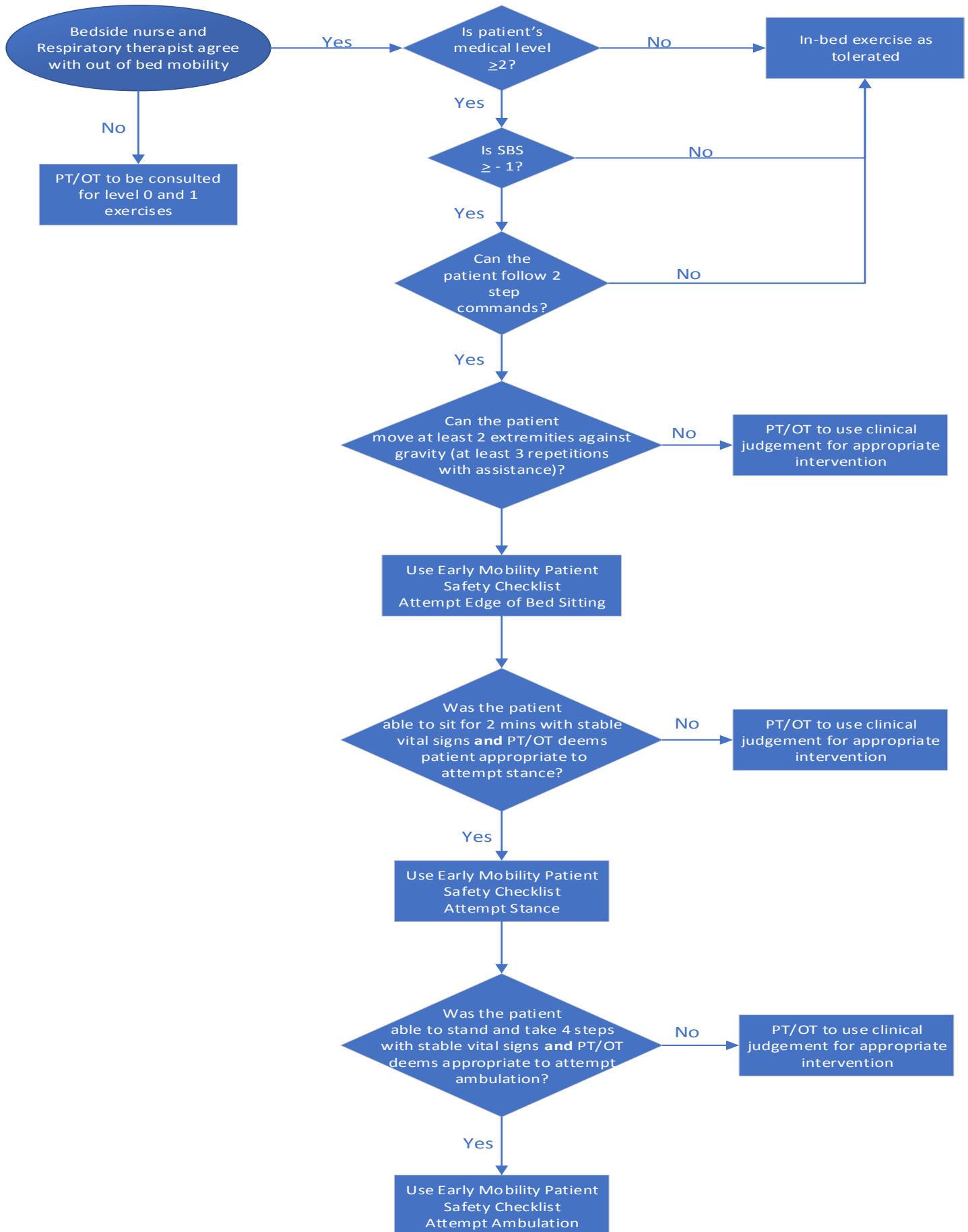

Supplement: Supplementary file 3 [file pqs-6-e369-s003.pdf]
