# Supplementary material for: Quality Improvement Methodology to Optimize Safe Early Mobility in a Pediatric Intensive Care Unit
Source: Pediatr Qual Saf. 2020 Dec 28;6(1):e369. doi: 10.1097/pq9.0000000000000369 (PMC7774997; doi:10.1097/pq9.0000000000000369)
Supplement: Supplementary file 4 [file pqs-6-e369-s004.pdf]

Contents of Early Mobility Cart

1. Draw sheets of different sizes
2. Backboard
3. Suction extension tubing
4. Monitor leads and Saturation probes
5. Portable monitor
6. Oxygen tubing and connector
7. Oxygen Flowmeter
8. End-tidal CO_2 detector (1 of each size)
9. Masks for Ambubag of various sizes
10. Christmas trees
11. Suction catheter kits (1 of each size)
12. Saline bullets
13. Tape
14. Scissors
15. Extra Velcro
16. Gait belt
17. Grip socks (1 of each size)
